# South-east Asian Zika virus strain linked to cluster of cases in Singapore, August 2016

**DOI:** 10.2807/1560-7917.ES.2016.21.38.30347

**Published:** 2016-09-22

**Authors:** Sebastian Maurer-Stroh, Tze-Minn Mak, Yi-Kai Ng, Shiau-Pheng Phuah, Roland G Huber, Jan K Marzinek, Daniel A Holdbrook, Raphael TC Lee, Lin Cui, Raymond TP Lin

**Affiliations:** 1 Bioinformatics Institute (BII), Agency for Science, Technology and Research (A*STAR), Singapore; 2 Department of Biological Sciences (DBS), National University of Singapore (NUS), Singapore; 3 School of Biological Sciences (SBS), Nanyang Technological University (NTU), Singapore; 4 National Public Health Laboratory (NPHL), Ministry of Health (MOH), CDC2, Singapore; 5 Department of Laboratory Medicine, National University Hospital, Singapore

**Keywords:** Singapore, vector-borne infections, Flaviviridae, viral infections, Zika virus, outbreaks, surveillance

## Abstract

Zika virus (ZIKV) is an ongoing global public health emergency with 70 countries and territories reporting evidence of ZIKV transmission since 2015. On 27 August 2016, Singapore reported its first case of local ZIKV transmission and identified an ongoing cluster. Here, we report the genome sequences of ZIKV strains from two cases and find through phylogenetic analysis that these strains form an earlier branch distinct from the recent large outbreak in the Americas.

## Outbreak detection

On 22 August 2016, the Ministry of Health (MOH), Singapore, was informed by a general practitioner of a spate of cases presenting with non-specific symptoms, including rash, polyarthralgia and low grade fever. In addition, a number of cases had mild conjunctivitis. Four days later, one of the cases was referred to the Communicable Disease Centre, Tan Tock Seng Hospital. The patient’s blood and urine samples tested positive for Zika virus (ZIKV), and the blood was negative for both dengue and chikungunya viruses by polymerase chain reaction (PCR). Since the patient had no travel history within the past month, the MOH announced the first locally transmitted ZIKV infection on 27 August, after laboratory confirmation on a second set of blood and urine specimens. For the purpose of outbreak investigation and public health measures, MOH then directed the collection of clinical specimens from cases working or living in the surrounding areas, with fever (>37.8°C), rash, conjunctivitis and/or joint pain in the preceding two weeks. All clinical information and samples pertaining to the outbreak investigation were collected under the provisions of the Infectious Diseases Act in Singapore [1].

## Genome sequencing of outbreak samples

A total of 153 individuals fitting the case definition were tested for ZIKV on 27 and 28 August, of which, only 56 cases were confirmed positive by real-time PCR assay [2]. Respective samples from two cases, ZKA-16–097 and ZKA-16–291, were selected based on their high viral titre (cycle threshold (Ct) values 21.7 and 24.6). Viral RNA was extracted from urine samples using the QIAamp viral RNA kit (Qiagen) and a series of overlapping reverse transcription (RT)-PCR reactions were performed using one-step Superscript III/Hi-Fidelity platinum Taq polymerase (Thermo Fisher). The primers were designed to target conserved regions among ZIKV whole genome sequences of both Asian and African lineages that were available on GenBank [3]. DNA bands of the predicted sizes were purified and sequenced by Sanger sequencing. Raw sequences were aligned and edited using CLC workbench. The consensus sequences for ZKA-16–097 and ZKA-16-291 were submitted to GenBank under the accession numbers KX813683 and KX827309, respectively.

## Background on Zika virus

ZIKV is a mosquito-borne single-stranded positive-sense RNA virus belonging to the *Flaviviridae* family. First isolated in 1947 from a sentinel rhesus monkey in Uganda [4], ZIKV circulated enzoonotically within Africa and equatorial Asia as two distinct lineages: the African and Asian lineage [5]. Prior to 2007, only 14 sporadic human infections, confined to Africa and equatorial Asia, were documented [6]. Since then, three outbreaks of ZIKV belonging to the Asian lineage have ensued: in 2007, on Yap island within the Federated States of Micronesia, in 2013 and 2014, within the French Polynesian islands and most notably, the current large outbreak in the Americas which was first detected in Brazil in 2015. Unlike the Yap island outbreak which was characterised by cases with relatively mild dengue-like symptoms [7], the outbreaks in French Polynesia and Brazil coincided with an unusual rate of cerebral congenital anomalies, including microcephaly [8]. Since 2015, 70 countries and territories reported evidence of ZIKV transmission [9]. The magnitude of spread of the Asian lineage and its disease association prompted the World Health Organization to declare ZIKV as a Public Health Emergency of International Concern in 2016 [10].

## Phylogenetic analysis

**Figure 1:**
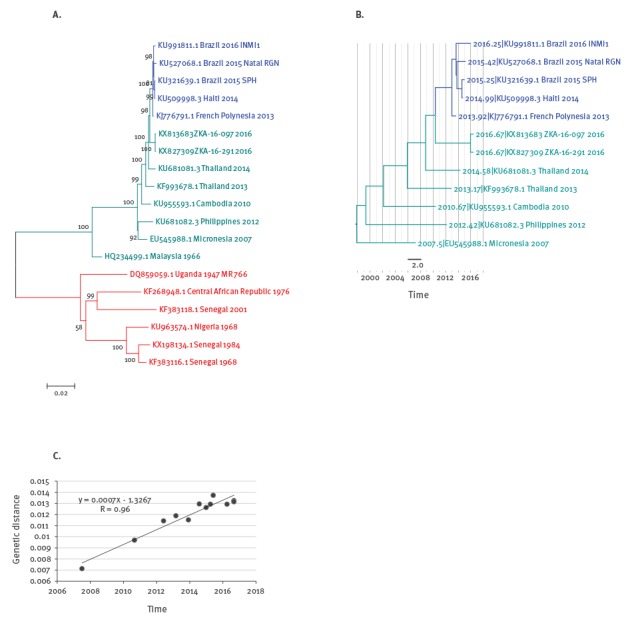
Phylogenetic trees with Zika virus genomes, 1947−2016

To gain further information on the ZIKV strains circulating within the current Singapore outbreak, the sequences recovered from the two cases were phylogenetically analysed. Complete ZIKV genomes available on GenBank were downloaded [3], and selected non-redundant representatives were aligned with the sequences from Singapore using Multiple Alignment using Fast Fourier Transform (MAFFT) [11]. The two Singapore sequences are 99.9% identical with only seven nucleotides different over 10,272 bases in the coding region. A maximum likelihood (ML) phylogenetic tree was created in Molecular Evolutionary Genetics Analysis (MEGA) [12] using the Tamura–Nei model with gamma distributed rate differences (5 categories, including invariant) and 1,000 bootstrap step validation. The phylogeny (Figure 1A) clearly shows the separate African and Asian lineages. Within the Asian lineage, the two sequences found in Singapore form a distinct branch, which stems from an ancestral node separating this branch from the tight cluster of strains from French Polynesia (2013), Haiti (2014) and Brazil (2015-2016) with 98% bootstrap support. The preceding shared ancestral node is with a strain circulating in Thailand in 2014 with 100% bootstrap support. Consequently, the viruses detected in Singapore evolutionarily arose in parallel to the large tight cluster of recent strains in South and Central America indicating that the two cases studied here were not infected by viruses imported from the Americas but rather by representatives of the Asian lineage already circulating in south-east Asia. The same clustering can be shown using other tree building methods and parameters (Bayesian: Figure 1B; neighbour joining, uniform rates, HKY model: data not shown).

A) Maximum likelihood phylogenetic tree of representatives from the African lineage coloured red and Asian lineage coloured blue or cyan. The tight cluster with strains from French Polynesia and the Americas is in blue and strains from south-east Asia are in cyan. The scale beneath the tree represents the number of substitutions per site. B) Time-resolved Bayesian tree of recent Asian lineage. The scale beneath the tree represents the years of divergence. C) Correlation of sampling date with phylogenetic distance for the recent Asian lineage.

Furthermore, in the tree, the long branch of the two new south-east Asian viruses found in this study suggests undetected evolution for several years. In order to estimate the time of divergence from the last common ancestor with the later cluster from French Polynesia and the Americas, we used a phylogenetic molecular clock approach through the Bayesian Evolutionary Analysis Sampling Trees (BEAST) package [13]. We first confirmed that the measured genetic distance within our set of the recent Asian lineage correlates well with the sample dates (Figure 1C) and created a time-resolved Bayesian tree with parameters previously established for ZIKV (strict clock model, Bayesian skyline, generalised time-reversible (GTR) [14]). The resulting tree (Figure 1B) is consistent with the clustering described in the ML tree and the time of the last common ancestor of the new south-east Asian strains and the French Polynesia/Americas cluster is estimated as 6.2 years ago (95% highest posterior density interval: 4.58–8.15 years) suggesting the observed clades diverged from each other around early 2010. Given this long unsampled evolution, future studies should establish possible reservoirs and circulation in the region.

## Conclusions from sequence and structure analysis

**Figure 2:**
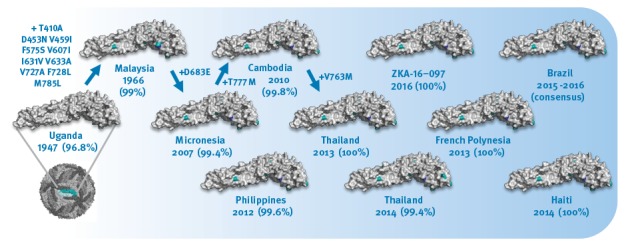
Mutations on the envelope (E) protein surface of different Zika virus strains

This study shows that there are still multiple ZIKV strains in circulation globally in 2016 which raises the question on their antigenic diversity or similarity for vaccine development. Therefore, we systematically mapped the outer surface envelope (E) protein changes from 1947 onwards across the whole Asian lineage (Figure 2).

Mutated residues in the Asian strain of Zika virus relative to the reference strain MR766 are indicated in cyan. Amino-acid residue numbers are according to polyprotein numbering. The highly conserved glycosylation site at N444 is indicated in blue. The schematic in the bottom left illustrates how the E proteins are arranged in a set of dimeric, trimeric, and pentameric elements to form a pseudo-icosahedral structure on the viral surface. 3D homology models were created using MODELLER [15] based on PDB:5IZ7 [16] and visualised with the PyMOL Molecular Graphics System (Version 1.8 Schrödinger, LLC.). The percent identity of each depicted strain’s E protein relative to the consensus of the Brazil 2015 sequences ranges from 96.8 to 100% and is indicated in parentheses.

At least within the Asian lineage the antigenic protein E surface is highly conserved and homogeneous with, from 2007 onwards, typical identities from 99.4% to 100% (or 3 to zero mutations) relative to the consensus of recent strains from Brazil. Moreover all of the E proteins in this lineage contain the typical N-glycosylation site (N444 in polyprotein numbering which is N154 in the E protein). The lack of surface mutation drift over the past 50 years (99% identity between Malaysia 1966 and consensus of Brazil 2015) also suggests that immune pressure on the E protein has not been a dominant factor in the Asian lineages’ virus fitness and evolution so far. While the highly similar surface E proteins of the different strains in the Asian lineage should facilitate global vaccine development it is too early to judge if the strain linked to the Singapore outbreak would show any different disease characteristics from the one in French Polynesia and the Americas. Further studies are necessary and underway in Singapore to collect more data.
